# MazEF-rifampicin interaction suggests a mechanism for rifampicin induced inhibition of persisters

**DOI:** 10.1186/s12860-020-00316-8

**Published:** 2020-10-27

**Authors:** Cyrus Alexander, Ankeeta Guru, Pinkilata Pradhan, Sunanda Mallick, Nimai Charan Mahanandia, Bharat Bhusan Subudhi, Tushar Kant Beuria

**Affiliations:** 1 Infectious Disease Biology, Institute of Life Sciences, Nalco Square, Bhubaneswar, India; 2 Manipal Academy of Higher Education, Manipal, Karnataka 576104 India; 3 Regional Centre for Biotechnology, Faridabad, Haryana 121001 India; 4 Animal Biotechnology Centre, National Dairy Research Institute, Harya, Karnal, – 132001 India; 5 Drug Development and Analysis, School of Pharmaceutical Sciences, Siksha ‘O’ Anusandhan Deemed to be University, Bhubaneswar, India

**Keywords:** TA system, MazEF, Rifampicin, Bacterial persistence

## Abstract

**Background:**

Persistence is a natural phenomenon whereby a subset of a population of isogenic bacteria either grow slow or become dormant conferring them with the ability to withstand various stresses including antibiotics. In a clinical setting bacterial persistence often leads to the recalcitrance of various infections increasing the treatment time and cost. Additionally, some studies also indicate that persistence can also pave way for the emergence of resistant strains. In a laboratory setting this persistent phenotype is enriched in nutritionally deprived environments. Consequently, in a batch culture the late stationary phase is enriched with persistent bacteria. The mechanism of persister cell formation and its regulation is not well understood. Toxin-antitoxin (TA) systems have been implicated to be responsible for bacterial persistence and rifampicin is used to treat highly persistent bacterial strains. The current study tries to explore a possible interaction between rifampicin and the MazEF TA system that furthers the former’s success rate in treating persistent bacteria.

**Results:**

In the current study we found that the population of bacteria in the death phase of a batch culture consists of metabolically inactive live cells resembling persisters, which showed higher membrane depolarization as compared to the log phase bacteria. We also observed an increase in the expression of the MazEF TA modules in this phase. Since rifampicin is used to kill the persisters, we assessed the interaction of rifampicin with MazEF complex. We showed that rifampicin moderately interacts with MazEF complex with 1:1 stoichiometry.

**Conclusion:**

Our study suggests that the interaction of rifampicin with MazEF complex might play an important role in inhibition of persisters.

**Supplementary information:**

The online version contains supplementary material available at 10.1186/s12860-020-00316-8.

## Background

Persisters represent a subset of a population of isogenic bacteria that are characterized by reduced or no growth, thereby helping the bacterial population tide over stressful situations including antibiotics [1]. Upon removal of the stress, these persister cells can go back to their normal growth and functioning without any acquired resistance. Whether they stochastically exist in the population as an insurance or is an induced phenotype to the stress, they play a key role in re-establishing many infections [2]. Apart from antibiotics, lack of nutrients is a major stress for the exponentially growing bacteria [3]. The number of persisters increase significantly during the late log and stationary phase when there is lack of nutrients [4, 5]. In a batch culture, the late stationary phase cultures showed increased persistence to various antibiotics which suggests that the late stationary phase bacteria behave as natural persisters. Although the mechanism of persister formation is not fully understood, the link between toxin–antitoxin (TA) modules and persisters formation is well recognized [6]. TA systems are ubiquitously present in all prokaryotes, which regulate several aspects of cell growth and survival strategies. In prokaryotes, TA systems consists of a stable toxin and a labile antitoxin. One of the well- studied TA modules in *E.coli* is the MazEF system. MazEF is a Type II TA system where MazE, an antitoxin, neutralizes MazF toxin, an endoribonuclease. Studies suggest that MazEF plays a significant role in bacterial persistence. Although the deletion or the over expression of the MazEF does not induce persister formation, [7, 8] the over expression of MazF alone induces the persister formation [7, 9]. Similarly, ClpP and Lon proteases, which degrade MazE, when deleted led to the decrease in persister population [9].

Bacterial persistence makes cells antibiotic tolerant which is a threat for disease control. This phenomenon has been widely studied in case of *Mycobacterium tuberculosis* where persisters comprise a larger population [10]. Rifampicin, a semi-synthetic broad-spectrum antibiotic, is used to treat persistent mycobacteria. It is recently reported that high doses of rifampicin not only clears the persisters and shortens the treatment time, but also prevents the relapse of tuberculosis [11, 12]. The above observations led us to investigate the correlation between the stationary phase persisters with MazEF expression and the effects of rifampicin. In this study we showed that MazEF expression is induced during the late phase of bacterial growth and rifampicin directly interacts with MazEF complex. For the first time we showed the interaction of rifampicin with a TA module.

## Results

### *E. coli* death phase population consists of live cells that are metabolically inactive

**Fig. 1:**
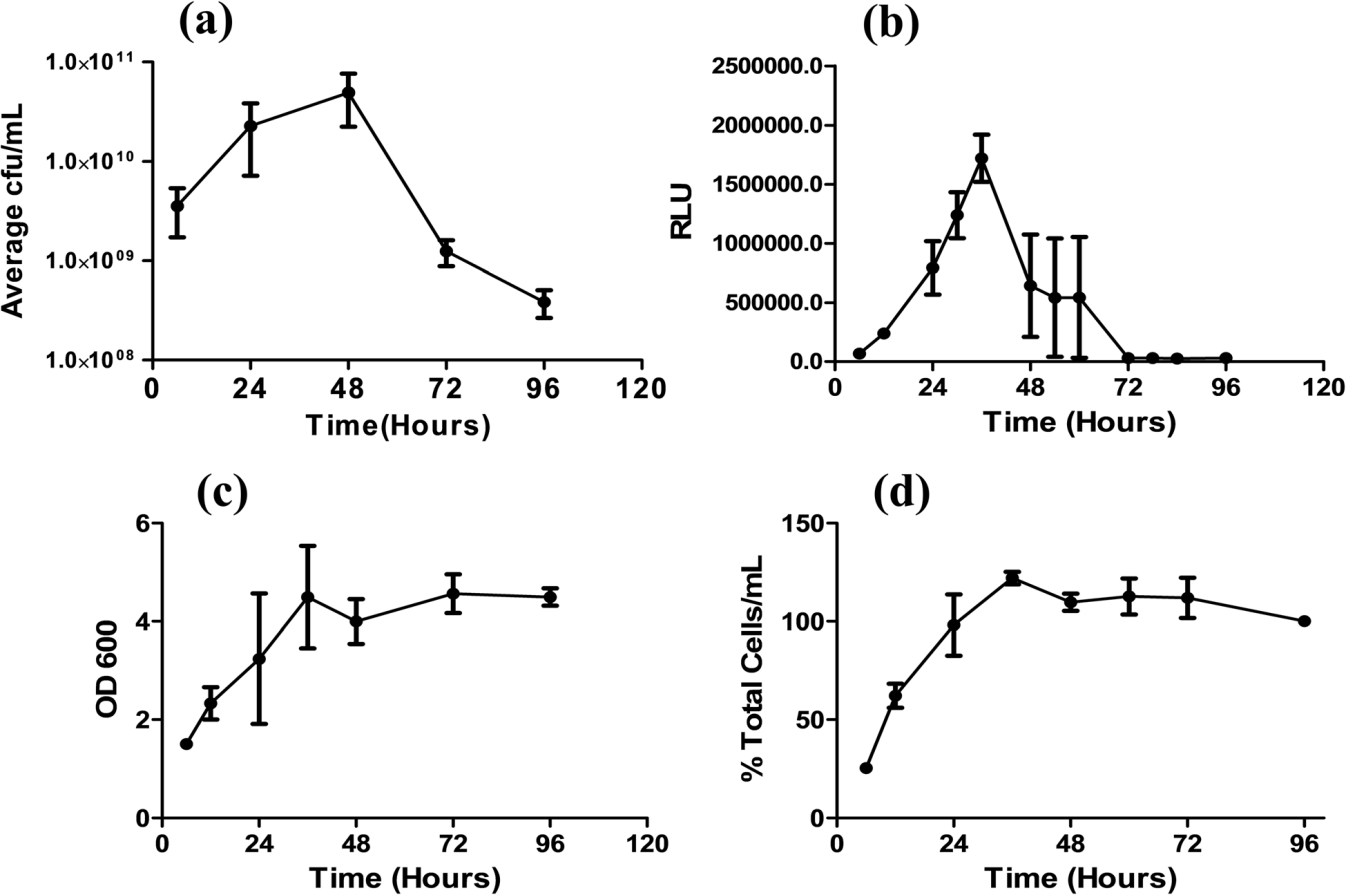
Drop in Viability and Metabolic Activity in a Closed Culture. *E.coli* was grown as mentioned earlier and samples were collected at various time point. Viable cell counts and metabolic activity were determined at different time points. Figures shows the viable cell counts (Panel **a**) and the metabolic activity (Panel **b**) of the bacteria at different time points. Panel **c** and **d** shows the relative cell numbers at different time points determined using OD_600_ and flow cytometry techniques respectively. All experiments were repeated three times and the graphs represent mean ± SEM

**Fig. 2:**
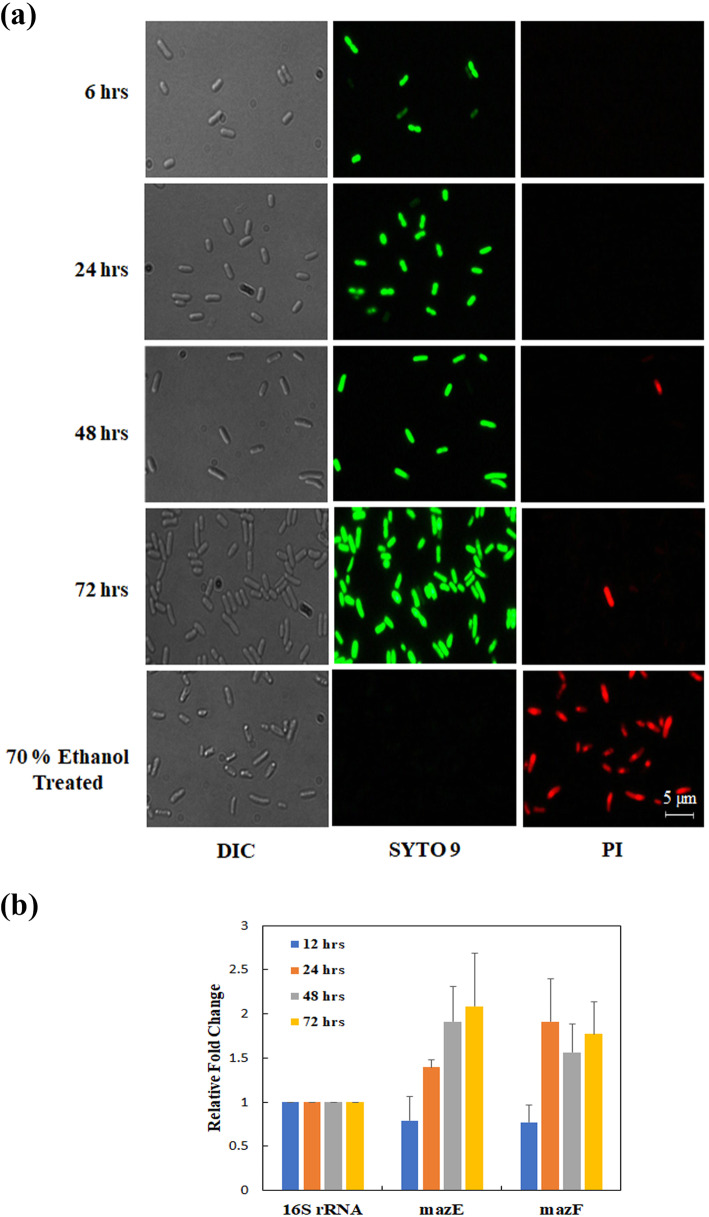
Live dead staining of bacteria. The live dead staining of *E. coli* at different time points was performed using live dead assay kit and imaging was performed using Olympus BX51 fluorescent microscope (Panel **a**). The left pane shows the total cells (DIC images), middle pane shows the live cells (green) and the right pane shows the dead cells (red). Expression of MazEF system was monitored at different time points for *E. coli* growing in a closed culture using qRT-PCR*.* Expressions of 16S-rRNA gene was used as control (Panel **b**). Graphs represents mean + SEM of qRT-PCR data of three independent experiments

In a batch culture, a typical bacterial growth curve consists of 5 distinct phases, i.e., lag phase, exponential phase, stationary phase, death phase and finally followed by a long-term stationary phase that is maintained for years [13]. The death phase in the growth curve has been considered for a long time as a stochastic event. When the cultural environment can no longer support the growth due to its limited resources in terms of nutrition, space and steady build-up of toxic metabolites, it causes cell death. When we examined cells from different stages of the growth curve, we observed a sharp decrease in the ability of *E.coli* to form colonies by 72 h (Fig. 1a). However, we did not notice any substantial increase in the dead cell population (Fig. 2a). We observed that by 72 h there was an approximate 95% drop in the ability of *E. coli* to grow in a LB agar plate (Fig. 1a). Whereas, when the same population was observed under microscope after staining with a live-dead stain, almost all the cells stained green, showing live cells (Fig. 2a). To further negate that the decrease in the colony forming units (cfu) was not due to the decrease in the actual cell numbers, we calculated the total number of cells by measuring the scattering at 600 nm (OD_600_) and counting the number of cells using a flow cytometer. The results showed an initial increase in the total relative cell counts till 40 h which remained unchanged until 96 h (Fig. 1c & d). We also found that the steady state *E. coli* was not affected by ampicillin, whereas, can be inhibited by rifampicin (Fig. S1). We determined the metabolic activity of the bacterial cells at various time points (Fig. 1b). The result showed an increase in the metabolically active cells until 40 h, which then decreased to near zero by 72 h. This data correlated with the cfu count data. Hence, we conclude that the death phase population of the *E.coli* growing in a batch culture mainly consisted of live cells with reduced metabolic activity, and have lost their ability to grow on solid LB agar plates.

### *E. coli* death phase population does not show hallmarks of apoptosis

**Fig. 3:**
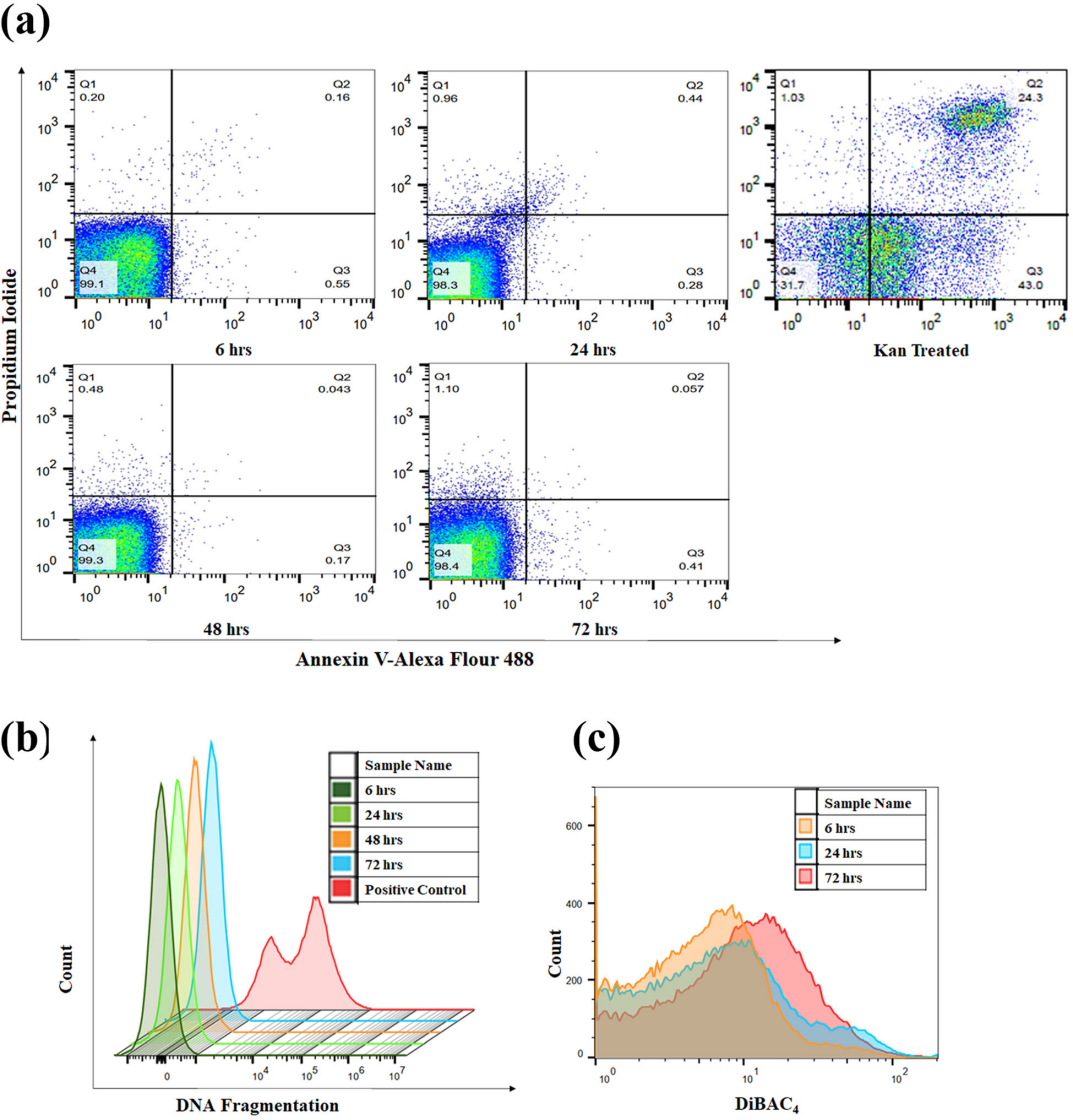
*E. coli* death phase does not show apoptotic hall marks. Annexin V/PI was used to determine whether apoptotic like death occurred during the death phase of the bacterial growth curve. *E. coli* at different time points of the growth curve showed no change in the Annexin V/PI labelled cells, whereas, Kanamycin treated showed increased number of Annexin V/PI labelled cells (Panels **a**). DNA fragmentation pattern (Panel **b**) and membrane depolarization (Panel **c**) are shown for cells at different stages of growth in a batch culture. All figures are representative images of three independent experiments

Historically the death phase in a batch culture has been considered to be mainly comprised of dead or dying cells. Due to nutrient and space limitations the cells can no longer grow and eventually stochastically die [14]. However, recent evidences suggest that programmed cell death may be a viable strategy in prokaryotes [15–17]. So, with this initial assumption we wanted to shed light on the mode of death during bacterial death phase. To prove this, we checked for the presence of two well characterized apoptotic markers i.e. Phosphatidylserine (PS) exposure and DNA fragmentation, at various stages of growth in a batch culture. It has been shown earlier that bacteria when exposed to antibiotics test positive for these markers showing apoptotic like death [18]. However, the late stationary phase cells and the death phase cells did not show any increase in either PS exposure or DNA fragmentation (Fig. 3a &b). Interestingly, we also did not see an increase in cells stained with propidium iodide which indicates the lack of dead cells in the bacterial death phase. These results corroborate our initial finding that the death phase population mainly comprise of live cells which have lost their ability to grow on LB agar plates. As a positive control we used kanamycin treated cells and they showed an increase in the propidium iodide and PS exposure (Fig. 3a).

### Loss in membrane polarity in the later stages of a batch culture indicates increase in persistent phenotype

The cell membrane in bacteria is a semipermeable membrane that protects the cell from many outer stresses including antibiotics. All living cells inherently and actively maintain a potential difference across its membrane thereby generating a membrane potential [19]. It is now known that the membrane potential is responsible for a wide range of signalling and processing. From pH homeostasis to cell division and even environmental sensing the bacterial membrane potential is dynamic tool [20]. Many antibacterial compounds achieve their goal by altering the membrane potential of the cell. Recent reports showed that some antibiotics can induce membrane depolarization and kill bacteria [21–23]. Alternatively, it is also shown that mild increases in membrane depolarization achieved by the cell itself in response to stresses can promote persister formation [24, 25]. We observed that *E.coli* grown in a batch culture tend to mildly increase its membrane potential in the whole population at different time points (6 h – 72 h) (Fig. 3c). This supported the idea that the persister formation in steady state and death phase might have increased due to change in membrane potential.

### Expression of the MazEF-TA modules increases overtime in a batch culture of the *E. coli*

The role of the TA modules in the bacteria is highly debatable. There are reports that suggest TA modules play an important role during bacterial programmed cell death, whereas, others advocate their roles during persister formation. Several type II TA modules are known to induce the persister formation in various bacteria. For example, overexpression of several toxins (i.e., TisB, HokB, etc.) reportedly increases the number of persister formation, whereas, deletion of the toxins decrease the number of persistent cells [26–28]. MazEF is one of the TA module that has been extensively studied and several reports support their role in the persister formation in different bacteria [9, 29, 30]. In *E. coli*, the MazF expression leads to growth arrest and enhance its survivability against various stresses [9, 31]. To understand the role of different TA modules during the death phase of the *E. coli*, we quantified the expression of MazEF along with five other Type-II TA modules (ChpBK/S, HicAB, MsqRA, RelEB, YoeB/YefM) using qRT-PCR. Our result showed that the expression of all the tested TA modules increased with increasing time (Fig. 2b & Fig. S2). Compared to the log phase (6 h) bacteria, the amount of MazEF increased nearly 2 folds in the steady state (48 h) and the death phase (72 h). Compared to the log phase, the number of persisters are reported to be higher during the steady state and the death phase of bacteria. Thus, our finding suggests that the higher MazEF expression may be related to higher persister formation in *E. coli*. As the MazEF TA system is well regarded as one of the factors responsible for the persister formation, we further focused our study to understand its interaction with rifampicin, a persister modulator.

### Rifampicin directly interacts with the MazEF complex

**Fig. 4:**
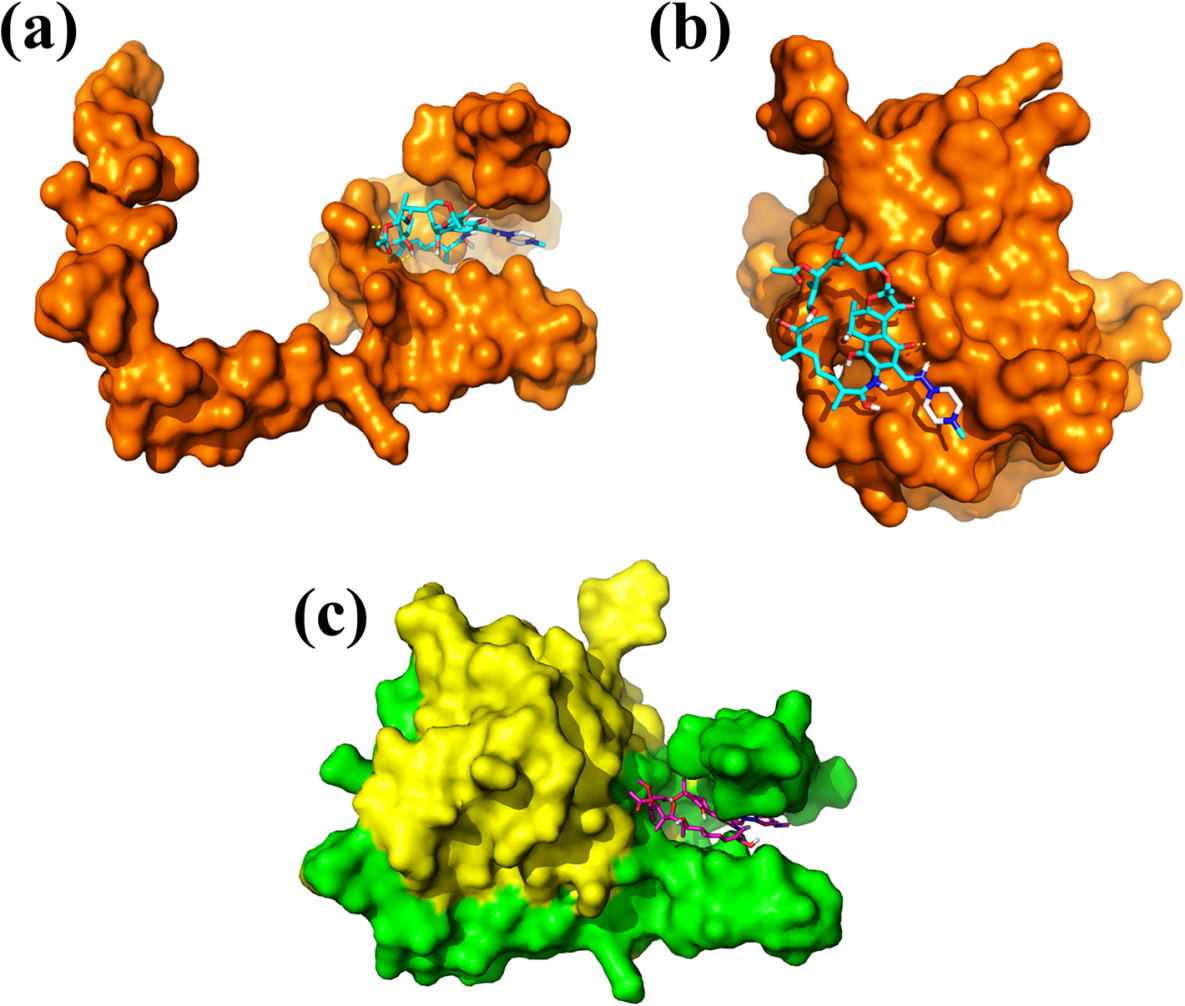
In silico and in vitro interaction of MazE/F with Rifampicin. Interaction of MazE/ MazF and MazEF with 800 molecules from FDA approved drug library was performed in silico using molecular docking. The figure shows that rifampicin interacts deep in the cavity with MazE (Panel **a**) and MazEF complex (Panel **c**) and not with MazF alone (Panel **b**)

Persisters can tolerate antibiotics not by acquiring any resistance, but through slowing down their metabolism. The activation of MazEF-TA module increases the number of persisters, whereas, bacteria lacking MazF becomes more susceptible to antibiotics [27, 30]. These observations suggested that targeting MazEF may provide a clue to target the persisters. To identify the molecules that can interact with the MazEF complex and inhibit bacterial growth, we performed molecular docking of MazEF complex, MazE or MazF with molecules from an FDA approved drug library containing 800 drug molecules. The 10 best ranked drugs against MazE, and MazF are shown in (Supplementary Table 1). Further analysis of the molecular docking data revealed that rifampicin has higher affinity (− 8.3 Kcal/mol) for MazE structure than MazF (− 6.2 Kcal/mol). In agreement with this data, rifampicin was found to bind in the deep pocket of MazE (Fig. 4a), whereas, for MazF it shows interaction on the surface (Fig. 4b). The molecular docking of rifampicin was carried out against the MazEF complex (PDB ID: 1UB4, chain A and C). As shown in the figure, rifampicin is predicted to preferentially interact in the same cavity of MazE (Fig. 4c). The *in-silico* data suggested that among the screened molecules rifampicin has a strong affinity against MazEF complex. Rifampicin is an antibiotic used for treatment of tuberculosis where persistence is a major problem. It is known to induce antibiotic tolerance in mycobacteria and higher dose can kill the persisters and reduce the duration of the treatment [11, 32].

**Fig. 5:**
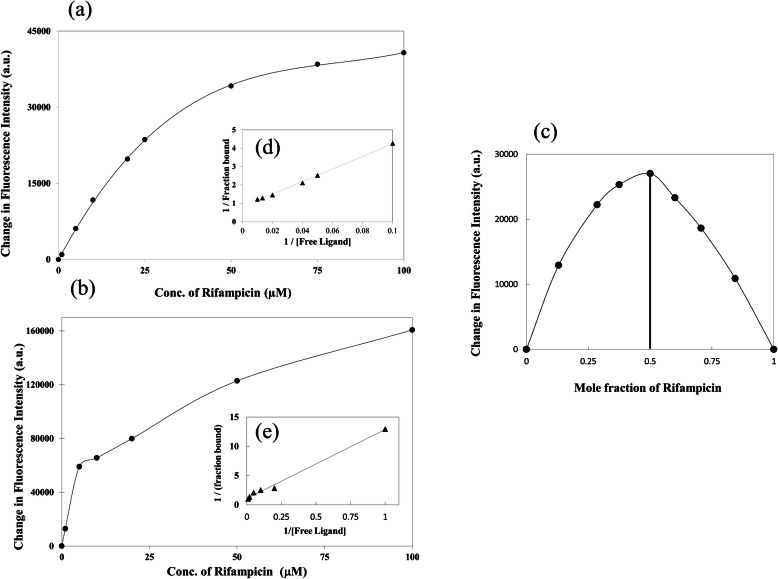
In vitro interaction of MazEF with Rifampicin. Panel **a** shows the change in intrinsic tryptophan fluorescence intensity with increasing concentrations of rifampicin. Panel **b** shows the change in Bis-ANS fluorescence intensity with increasing rifampicin concentration. The inset **d** and **e** in Panel **a** and **b** shows the respective double reciprocal plots. Panel **c** is the Job’s plot showing the 1:1 stoichiometry for rifampicin binding to MazEF. All the experiments were performed in triplicates

To confirm the direct interaction of MazEF with rifampicin, we purified the MazEF complex (Fig. S3) and performed the interaction studies with rifampicin using fluorescence binding assay. We used two different ways to determine the interaction between MazEF and rifampicin. First, intrinsic tryptophan fluorescence was used to determine the MazEF-rifampicin interaction. The result showed that rifampicin interacts moderately with MazEF complex with a dissociation constant of 42 ± 8 μM (Fig. 5a). Second, MazEF was labelled with a hydrophobic fluorescent dye Bis-ANS and the change in Bis-ANS fluorescence on addition of rifampicin was used to determine the MazEF-rifampicin interaction. Our result showed a dissociation constant of 12.9 ± 4.7 μM for MazEF-rifampicin complex (Fig. 5b). Both the results indicated moderate interaction of rifampicin to the MazEF complex. We further determined the stoichiometry of MazEF-rifampicin complex using Job’s continuous variation method (Fig. 5c). Our result showed that rifampicin interacts with MazEF with a stoichiometry of 1:1. To verify that the interaction of rifampicin with MazEF complex is a specific one, we determined the interaction of ampicillin with MazEF complex. Ampicillin did not affect the persister formation and was previously used by several researchers to kill the normal bacteria and enrich the persisters. Interestingly, our *in-silico* data showed that ampicillin does not interact with the MazE. The affinity of ampicillin with MazE was calculated to be − 6.4 Kcal/mol which is much lower as compared to the interaction between rifampicin-MazEF complex (− 8.3 Kcal/mol). Similarly, fluorescence binding assay did not show any significant change in the fluorescence of MazEF complex when titrated against ampicillin, suggesting that ampicillin does not interact with MazEF complex (data not shown).

## Discussion and conclusion

Although bacterial persistence is one of the main reasons for recalcitrance of various infections, its importance was acknowledged on par with bacterial resistance relatively recently [33]. The major difference between antibacterial resistance and persistence is that the antibacterial resistance is caused by a heritable trait that is spontaneously generated giving rise to mutants that can actively neutralize a given threat. While persistence represents a subset of the microbial population that is either dormant or slow growing there by ensuring that a part of the population survives a given catastrophic event [34]. On the return of favourable conditions these dormant bacteria can grow and re-establish the original population which is susceptible to the original stress. The mechanism by which isogenic bacteria achieve such a reversible persistent state is not fully understood. Yet evidences suggest that persistence may be the end result of stochastic activation of toxins from toxin-antitoxin systems present in microbes. The TA systems represent genetic loci that code for a protein toxin that can cause growth arrest by interfering with essential cellular processes, and a corresponding antitoxin that is co-expressed that neutralizes the toxin [35]. The antitoxin has a lower stability as compared to the toxin and consequently has a high turn-over rate. Conditions that prevent the antitoxin production or accelerate its degradation can thus activate the toxin. Similarly, conditions that favours antitoxin production, stability or the degradation of the toxin may prevent persister formation. The MazEF TA system is one of the well-studied Type II TA systems in *E.coli* and has been linked to cause persistence upon activation. The ectopic overexpression of MazF in *E.coli* causes growth arrest and this dormancy can be reversed by ectopically over-expressing the antitoxin MazE [7]. This reversibility of dormancy is time bound as beyond the “point of no-return” the toxin becomes too toxic for the cell [36]. In addition to antibacterial stresses, nutritional stress can also lead to the formation of persisters [3]. In stationary phase bacteria nearly 1% of the cells comprise of persisters which makes it difficult to be treated by any antibiotics [3]. Our study showed that bacteria present in the steady state or death phase in a batch culture majorly consisted of live but metabolically inactive cells, which have lost their ability to form colonies on nutrient agar plates. These bacterial population also did not show any apoptotic markers that are commonly shown by bacteria when treated with antibiotics. This suggests that the death phase cells from the bacterial culture are morphologically and functionally different from the dying bacterial cells treated with antibiotics. We also observed an increase in the MazEF TA modules in these late stationary phase and death phase bacteria. These bacterial populations were also resistant towards ampicillin, but sensitive to rifampicin suggesting their resemblance with the persisters (Fig. S1). Our *In-silico* and biochemical experiments showed that MazEF interacts directly with rifampicin, a commonly used antibiotic against persisters, with moderate affinity. We further showed that the stoichiometry of the MazEF-rifampicin complex is 1:1. It is currently known that rifampicin acts by interacting with bacterial DNA dependent RNA polymerase and inhibiting its function [37]. This interaction of rifampicin with RNA polymerase is very strong (Kd = 1 nM at 37 °C) [38] and thus a low amount of rifampicin should able to kill bacteria. Likewise, the recommended dosage of rifampicin is 10 mg/kg, however, this dosage is not sufficient to kill the persisters [11]. Several studies have suggested that MazEF complex plays a major role during persister formation [9]. In the presence of antibiotics or other stress, MazF expression induces reversible persister formation which can regrow once the antitoxin MazE is being synthesized and sequesters MazF [31]. Recent reports showed that high dose of rifampicin (100 μg/mL) is not only able to kill the persisters, but also reduces the treatment duration and prevents disease relapse in Mtb [11]. Our results showed that the rifampicin at this concentration interacts with MazEF complex. Overall, our study suggests that the interaction of rifampicin with MazEF might play a role to inhibit persister formation. However, a detailed study is required for a better understanding of the system.

## Methods

### Growth conditions and growth curve

The growth curve was monitored using standard procedure mentioned earlier [39]. Briefly, *E. coli* MG1655 (WT) was streak plated onto LB-agar nutrient plate and incubated at 37 °C for 12–15 h. A single colony was inoculated in 50 mL LB broth and grown overnight at 37 °C and 200 rpm. From the above culture 100 μL was transferred to fresh 250 mL of LB broth in 500 mL flasks and allowed to grow at 37 °C and 200 rpm. Samples were collected at various time points and were used for various experiments as described below. The total number of cells at each time points was monitored by OD_600_ and flow cytometric cell count. To determine the viable cell count, cells were spread on LB agar plates and incubated at 37 °C for 12–15 h. The lowest dilution with more than 30 colonies was considered for viable cell counts.

### Metabolic state of *E. coli* MG1655 at various stages of growth

*E.coli* MG1655 was grown in 250 mL LB-broth as mentioned earlier. Samples were collected at various time points and added to an opaque walled 96-well plate. An equal volume of BacTiter-Glo™ reagent was added as per manufacturer’s instructions, incubated for 5 min and luminescence was measured using an ELISA plate reader. The luminescence is directly proportional to the total ATP content which is representative of the metabolic state of cells.

### Live-dead assay and microscopy

*E.coli* MG1655 was grown in 250 mL LB-Broth as mentioned earlier. 1 mL samples were collected at various time points and centrifuged at 10,000×g for 10 min. All samples were washed with 0.85% NaCl twice and resuspended in the same and the OD_600_ was adjusted to 0.1. Again, all samples were diluted 1:10 with 0.85% NaCl to a final volume of 1 mL. To determine the live dead status of the culture, BacLight Bacterial viability kit (Invitrogen) was used. The kit uses SYTO9 (green) to visualize live cells and propidium iodide (red) to visualize the dead cells. A 1:1 mixture of SYTO9-PI reagent (3 μL) was added to 1 mL of diluted sample, incubated for 15 min at room temperature. Slides were prepared and samples were observed under fluorescence microscope (Olympus BX51) as described previously [40].

### Detection of Phosphatidylserine and membrane compromise

*E.coli* MG1655 was grown as stated above, samples were collected at various time points and washed twice with 1X PBS pH 7.2. The cells were then diluted in 1 X annexin V binding buffer to a concentration of one million cells/mL. Cells were stained by annexin V and propidium iodide as recommended by the kit manufacturer. The cells were incubated for 15 min in the dark and then acquired using BD FACS Calibur and results were analysed using FlowJo™ V 10 software.

### Membrane depolarization assay

*E.coli* MG1655 was grown as stated above and samples were collected at various time points. 20 μL of sample from each time point was added to 200 μL reaction mixture containing 0.85% NaCl and 20 μL of DiBAC_4_ (100 μg/mL + 0.5% Tween 20) and incubated at 25 °C for 30 min in the dark. The fluorescence intensity was determined using BD FACS Calibur and the results were analyzed using FlowJo™ V 10 software.

### DNA fragmentation assay

*E.coli* MG1655 was grown as mentioned earlier and samples were collected at various time points. The samples were fixed with 1% paraformaldehyde for 30 min on ice. They were then washed with 0.85% NaCl twice and resuspended in ice cold 70% ethanol and stored at − 20 °C till the day of experiment. To observe the DNA fragmentation profile during different stages of growth, samples were stained with APO-BrdU™ TUNEL assay kit and data acquired using Beckman Coulter Cytoflex-S and results were analysed using FlowJo™ V 10 software.

### Quantification of TA systems at various time points using qRT-PCR

RNA was isolated from the cells collected at different time points using Qiagen RNeasy RNA isolation kit and cDNA library was prepared. Then qRT-PCR was performed to quantify the relative abundance of mRNA of various toxins and antitoxins.

### In silico screening of MazEF interacting molecules

The structure of MazEF complex with PDB ID: 1UB4 was selected as the target. For MazE, and MazF, the chain A and C was used. Drugs from FDA approved library were screened against these targets using the Autodock Vina program and ranked based on their binding affinities for these targets. Rifampicin has good ranks against both targets and thus selected for molecular docking using the Autodock Vina program and analysed using the PyMol program [41].

### Purification of MazEF complex

Purification of MazEF complex was carried out using standard procedure with some modifications [42]. The detailed procedure is mentioned in Supplementary method section (Fig. S3, SM1).

### Fluorescence interaction studies

The interaction between MazEF and rifampicin was determined by fluorescence assay using the procedure mentioned earlier with few modifications [43]. We used both intrinsic tryptophan fluorescence and Bis-ANS labelling to determine the dissociation constant. First, purified MazEF (1 μM) was titrated against increasing concentrations of rifampicin (5–100 μM). The tryptophan emission fluorescence spectra (310–400 nm) was monitored for an excitation wavelength of 285 nm. The change in fluorescence intensity at 340 nm was used to determine the equilibrium dissociation constant using a double reciprocal plot. Second, MazEF was labelled with Bis-ANS by incubating the protein with 10 folds higher molar concentration of Bis-ANS dye in 50 mM Tris buffer, pH 8 containing 150 mM NaCl for 1 h at 4 °C. The reaction mixture was dialyzed overnight in 25 mM HEPES buffer (pH 7.4) containing 50 mM NaCl. The labelled MazEF (1 μM) was titrated against increasing concentrations of rifampicin (1–100 μM) and the Bis-ANS emission fluorescence spectra (400–600 nm) was monitored for an excitation wavelength of 380 nm. The change in fluorescence intensity at 495 nm was used to determine the equilibrium dissociation constant using a double reciprocal plot. The stoichiometry of MazEF and rifampicin was assessed using Job’s method of continuous variation. Then the change in Bis-ANS-MazEF fluorescence was observed with increasing the rifampicin concentration and decreasing the protein concentration while keeping the total concentration constant at 1 μM. This change in fluorescence intensity was used for Job’s plot.

## Supplementary information

Additional file 1: **SM1.** Method for Purification of MazEF protein complex. **SM2.** Method for Western Blotting for detection of (His)_6_MazE. **Fig. S1**. Effects of antibiotic on *E. coli* from different growth times. **Fig. S2**. Expression of TA systems at different time points. **Fig. S3**. Purified MazEF complex detected by SDS PAGE and Western Blotting. **Supplementary Table 1**. List of molecules interacted with MazE/ MazF in silico. (DOCX 2054 kb)

## Data Availability

The datasets used and/or analysed during the current study are available from the corresponding author on reasonable request.

## Abbreviations

TA: Toxin-antitoxin
WT: Wild-type
cfu: Colony forming units
PS: Phosphatidylserine
PI: Propidium iodide
