# Supplementary material for: MazEF-rifampicin interaction suggests a mechanism for rifampicin induced inhibition of persisters
Source: BMC Mol Cell Biol. 2020 Oct 27;21:73. doi: 10.1186/s12860-020-00316-8 (PMC7590665; doi:10.1186/s12860-020-00316-8)
Supplement: Supplementary file 1 — SM1. Method for Purification of MazEF protein complex. SM2. Method for Western Blotting for detection of (His)6MazE. Fig. S1. Effects of antibiotic on E. coli from different growth times. Fig. S2. Expression of TA systems at different time points. Fig. S3. Purified MazEF complex detected by SDS PAGE and Western Blotting. Supplementary Table 1. List of molecules interacted with MazE/ MazF in silico. (DOCX 2054 kb) [file 12860_2020_316_MOESM1_ESM.docx]

**Supplementary Materials:**

**Supplementary Methods.**

**SM1. Purification of MazEF protein complex:** Overnight starter culture of *E. coli* BL21 (DE3) with MazEF-pET28a plasmid is inoculated in 1L LB media containing kanamycin (50 μg/mL). The bacterial culture is then grown at 37 ºC till OD_600_ ~ 0.4. MazEF complex expression is induced by the addition of 1 mM IPTG and grown for another 4 hours at 30 °C. Cells were harvested by centrifugation at 8000 rpm. The pellet was re-suspended in 40 mL Lysis Buffer (50 mM Tris-Cl, 300 mM NaCl and 10 mM Imidazole, pH 8) containing 1 mM PMSF and protease inhibitor cocktail. Cells were lysed using Stansted pressure cell homogeniser and the lysate was centrifuged at 15,000 rpm for 30 min. Subsequent steps were performed at 4 ºC. The supernatant was incubated with prewashed 5 mL HisPur^TM^ Cobalt resin for 1 hour with gentle shaking. The protein complex trapped on the column was washed with 20 mL Wash Buffer (50 mM Tris-Cl pH 8, 300 mM NaCl and 20 mM Imidazole) and eluted in 15 mL elution buffer (50 mM, 300 mM NaCl, 250 mM Imidazole). The eluted fraction was dialysed overnight to remove NaCl and imidazole against 50 mM Tris buffer (pH 8) containing 150 mM NaCl. The purified fraction contains both MazE and MazF as the protein complex.

**SM2. Western Blotting for detection of (His)_6_MazE:** Purified MazEF complex was separated into its components on a 15 % SDS gel and then transferred to PVDF membrane. After blocking for 1 hr at RT with 5 % skimmed milk the membrane was incubated overnight at 4 ºC on a gel rocker with primary antibody (mouse monoclonal anti-polyhistidine diluted 1:20,000 in blocking buffer). After washing the membrane, it is incubated at room temperature on a gel rocker for 1 hr with secondary antibody (goat anti-mouse conjugated to horse radish peroxidase diluted 1:5,000 in blocking buffer). The membrane is then washed thrice and Pierce ECL Plus is used as the substrate for HRP and chemiluminescence is detected by Biorad ChemiDoc MP.

**Supplementary Figures.**


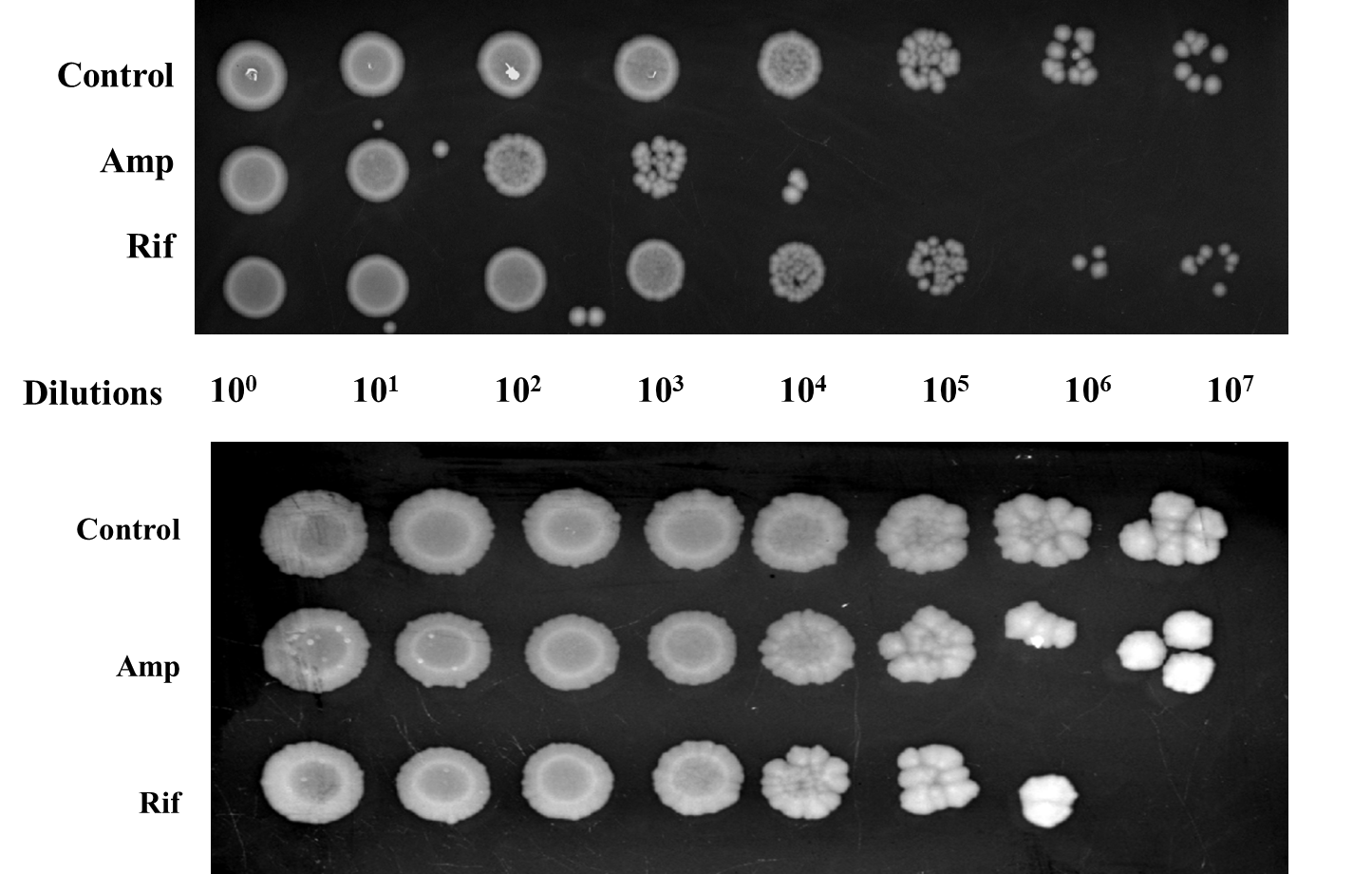


B

A

***Figure S1. Effects of antibiotic on E. coli from different growth times.*** *E. coli* cells were grown as stated above, cells were collected at 6 hrs (***Panel A***) or 48 hrs (***Panel B***) and treated with Ampicillin (80 μg/mL) or Rifampicin (125 μg/mL). The cells were then incubated for 3 hours at 37 ºC. After the dilution the cells were serially diluted with LB broth and 2 μL from each dilution were plated on a LB agar plate. The plates were incubated for 12-16 hours at 37 ºC and images were taken using BioRad Chemidoc.


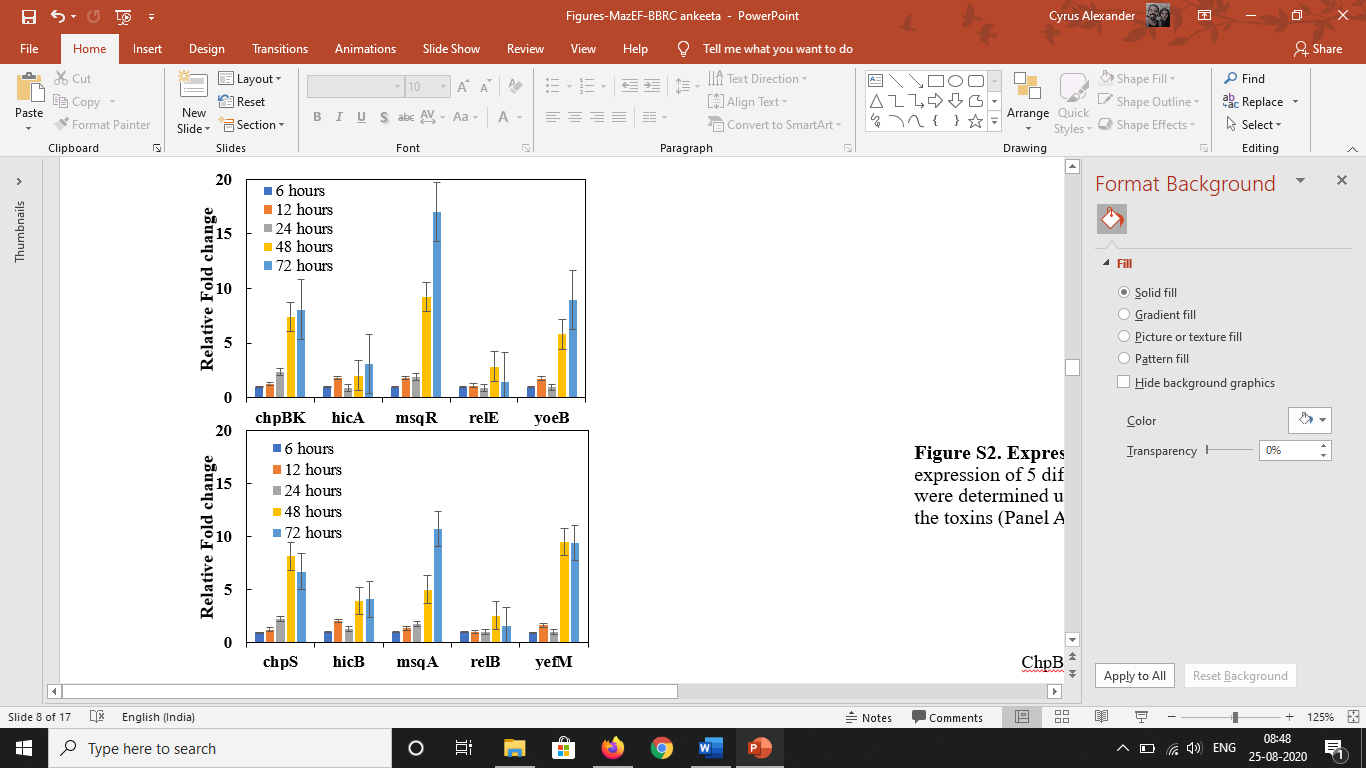


B

A

***Figure S2.* *Expression of TA systems at different time points.*** The expression of 5 different TA systems at different growth time points were determined using qRT-PCR. The figure shows the expression of the toxins (***Panel A***) and the antitoxins (***Panel B***). Three independent experiments were conducted and figures represent mean ± SEM.


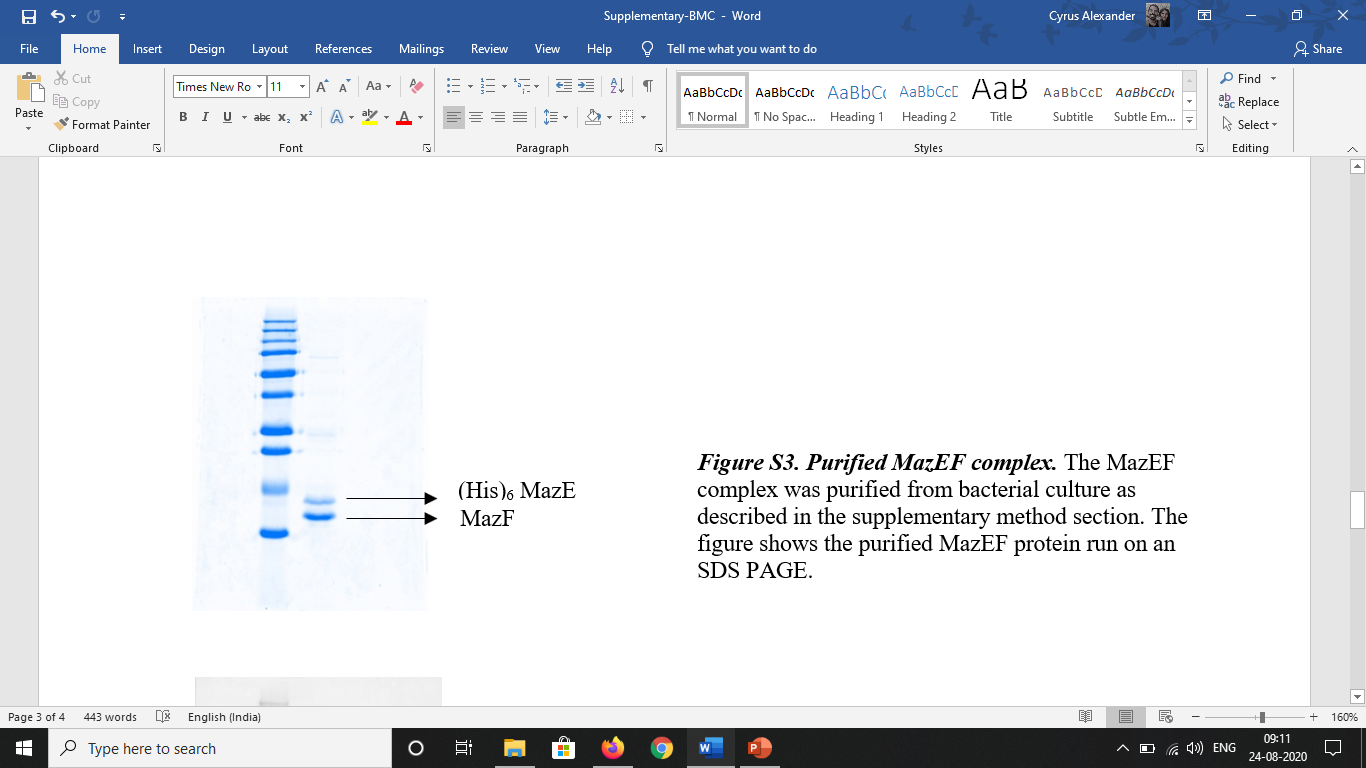

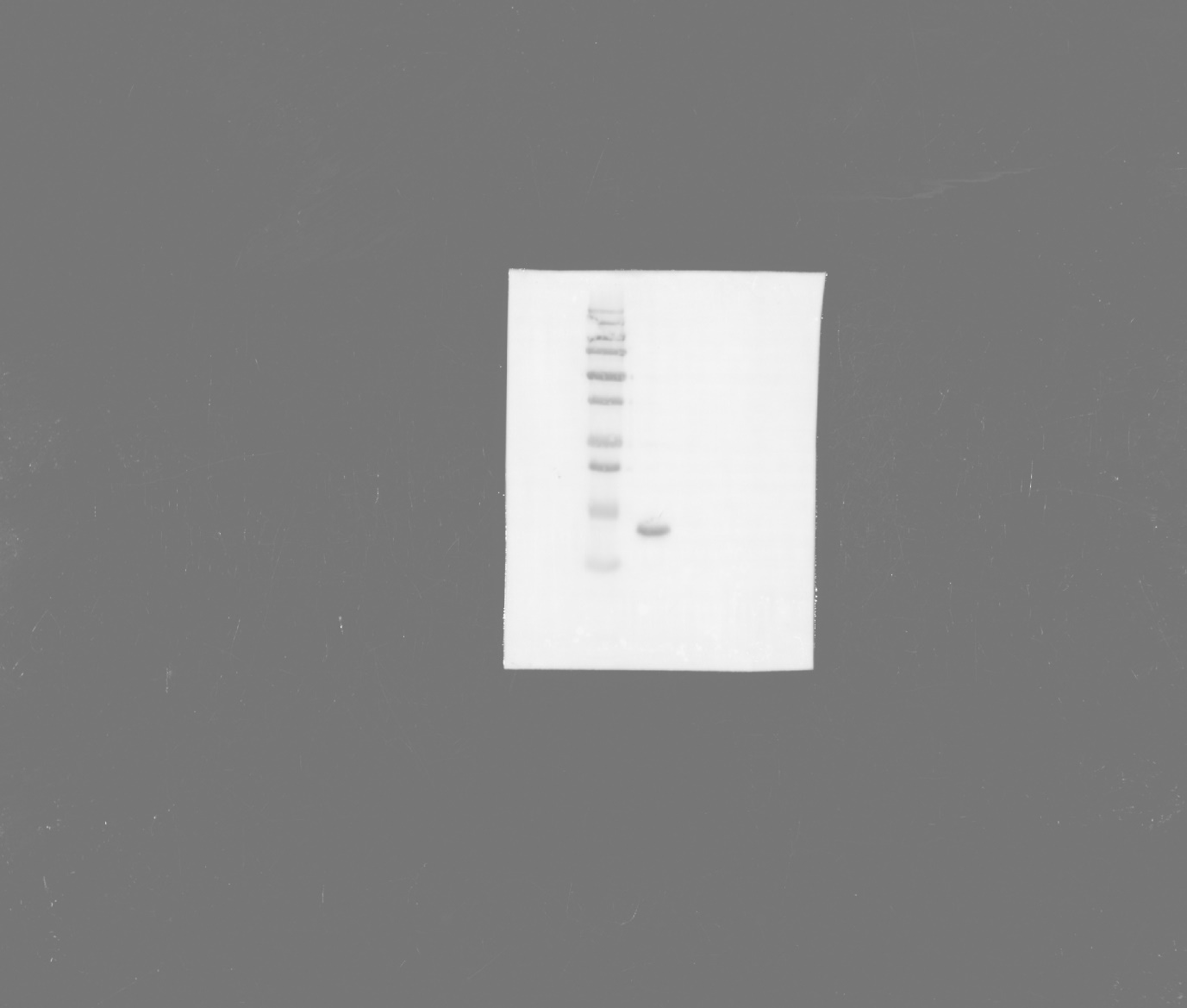


(His)_6_ MazE

B

A

***Figure S3.* *Purified MazEF complex.*** The MazEF complex was purified from bacterial culture as described in the supplementary method section. The figures show the purified MazEF protein run on a 15 % SDS PAGE (***Panel A***) and (His)6-MazE detected by anti-polyhistidine antibody using Western Blotting (***Panel B***).


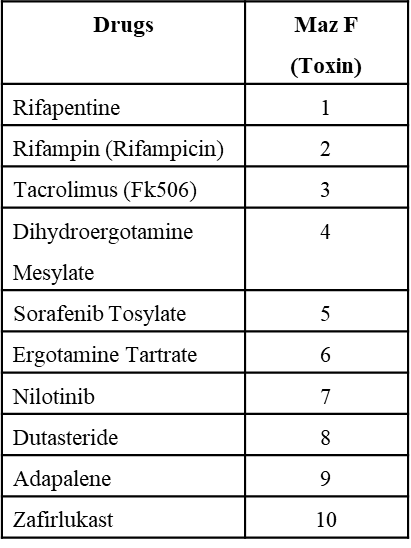

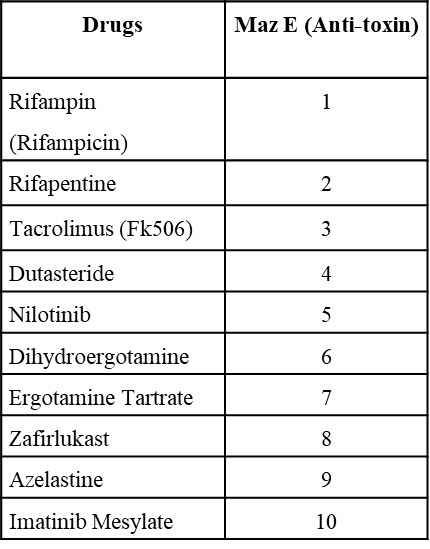


***Supplementary Table 1.* *List of molecules interacted with MazE/ MazF in silico.*** The structure of MazEF complex with PDB ID: 1UB4 was selected as the target. The drug molecules from FDA approved library were screened against these using the method described in the method section. The compounds were ranked based on their binding affinity (Kcal/mol). The 10 best ranked drugs against MazE, and MazF are shown in the tables.
